# The Temporal Lobe as a Symptomatogenic Zone in Medial Parietal Lobe Epilepsy

**DOI:** 10.3389/fneur.2022.804128

**Published:** 2022-03-15

**Authors:** Nadim Jaafar, Amar Bhatt, Alexandra Eid, Mohamad Z. Koubeissi

**Affiliations:** ^1^Department of Neurology, George Washington University, Washington, DC, United States; ^2^Rush Medical College, Rush University, Chicago, IL, United States

**Keywords:** temporal lobe epilepsy, parietal lobe epilepsy, epilepsy surgery, intractable epilepsy, cingulate gyrus, precuneus cortex

## Abstract

Some surgical failures after temporal lobe epilepsy surgery may be due to the presence of an extratemporal epileptogenic zone. Of particular interest is the medial parietal lobe due to its robust connectivity with mesial temporal structures. Seizures in that area may be clinically silent before propagating to the symptomatogenic temporal lobe. In this paper, we present an overview of the anatomical connectivity, semiology, radiology, electroencephalography, neuropsychology, and outcomes in medial parietal lobe epilepsy. We also present two illustrative cases of seizures originating from the precuneus and the posterior cingulate cortex. We conclude that the medial parietal lobe should be strongly considered for sampling by intracranial electrodes in individuals with nonlesional temporal lobe epilepsy, especially if scrutinizing the presurgical data produces discordant findings.

## Introduction

Presurgical evaluation of patients with pharmacoresistant temporal lobe epilepsy (TLE) integrates multimodal diagnostic tools with the aim of identifying the seizure onset zone. These often include a careful assessment of the seizure semiology, electroencephalography (scalp video-EEG), neuropsychology, and radiology (MRI, PET, SPECT). Further evaluation using invasive procedures such as stereotactic-EEG (sEEG) or subdural electrode implantation may be utilized if the non-invasive tests yield inconclusive or discordant results (1–3). Nevertheless, surgical failures in TLE are still encountered (4).

About 30–40% of patients do not achieve seizure freedom after TLE surgery (5–7). In some cases, this may be due to a cortically silent extratemporal seizure-onset zone from which seizures rapidly propagate to the temporal lobe, where they become clinically and electrographically manifest. These zones may be frontal, occipital, parietal, opercular, or insular. Other reasons for surgical failure include insufficient resection of epileptogenic zone, multifocal epilepsy, varied temporal lobe pathology (mesial and/or lateral TLE), and temporal-plus epilepsy (5).

Here, we review medial parietal lobe epilepsy (PLE) as a seizure onset zone with preferential propagation to the temporal lobe, potentially masquerading as TLE. We present two lesional cases of seizures originating in the precuneus (8) and the posterior cingulate cortex (PCC) (9), manifesting as TLE. Finally, we highlight the importance of having a multimodal integrated evaluation of the implicated anatomical connectivity, semiology, neuroimaging, neuropsychological testing, and electroencephalography to optimize surgical outcomes.

## Anatomy and Functional Connectivity

The posteromedial cortex of the parietal lobe, which comprises the precuneus/medial parietal cortex, posterior cingulate cortex (PCC), and retrosplenial cortex (10, 11) has bilateral reciprocal connections with the temporal lobe (12, 13). Cerebro-cerebral evoked potentials (CCEP), functional and anatomical imaging, and clinical and animal studies provide evidence for this connectivity. Specifically, the PCC has connections with both the mesial and lateral parietal and temporal cortices (14).

The precuneus is located medially in the superior portion of the parietal lobe between the primary somatosensory cortex and the visual cortex (cuneus) (15). It includes three heterogeneous entities, each with distinct connections to other cortical/subcortical structures. A study in macaque monkeys assessing connectivity of the precuneus has found that the anterior precuneus is extensively connected with sensorimotor structures such as the paracentral lobule, superior-parietal cortex, and motor cortex (12). The central precuneus has connections with cognitive and associative prefrontal/frontal cortical regions. Finally, the posterior precuneus is functionally associated with the visual cortex (12, 15). The precuneus forms the midline core of the default mode network, a network that is predominantly active during periods of inactivity (16–18). Other brain structures that are included in the default mode network are the PCC and the mesial temporal lobe (MTL). Thus, extensive functional connectivity between the precuneus and the MTL is evident in the resting state (10). Moreover, tractography data and post-mortem analysis have shown extensive projections from the posterior precuneus to the MTL, specifically, the anterior para-hippocampal gyrus (16). Seizures originating from the precuneus may be clinically silent or potentially cause some alteration of awareness (19, 20). When clinically silent, they may be mistaken as temporal onset seizures because the scalp-recorded ictal discharge and seizure semiology may be indistinguishable from TLE.

## Illustrative Case 1: (8)

This was a 31-year-old right-handed man with a remote history of neurocysticercosis. His seizures started at age 12 with left sided piloerection followed by a brief alteration of awareness. Years later, the piloerection ceased to be part of his seizures and he started having cephalic auras. The patient maintained a seizure frequency of 5–10 seizures per month despite being on numerous anti-seizure medications (ASMs).

**Figure 1:**
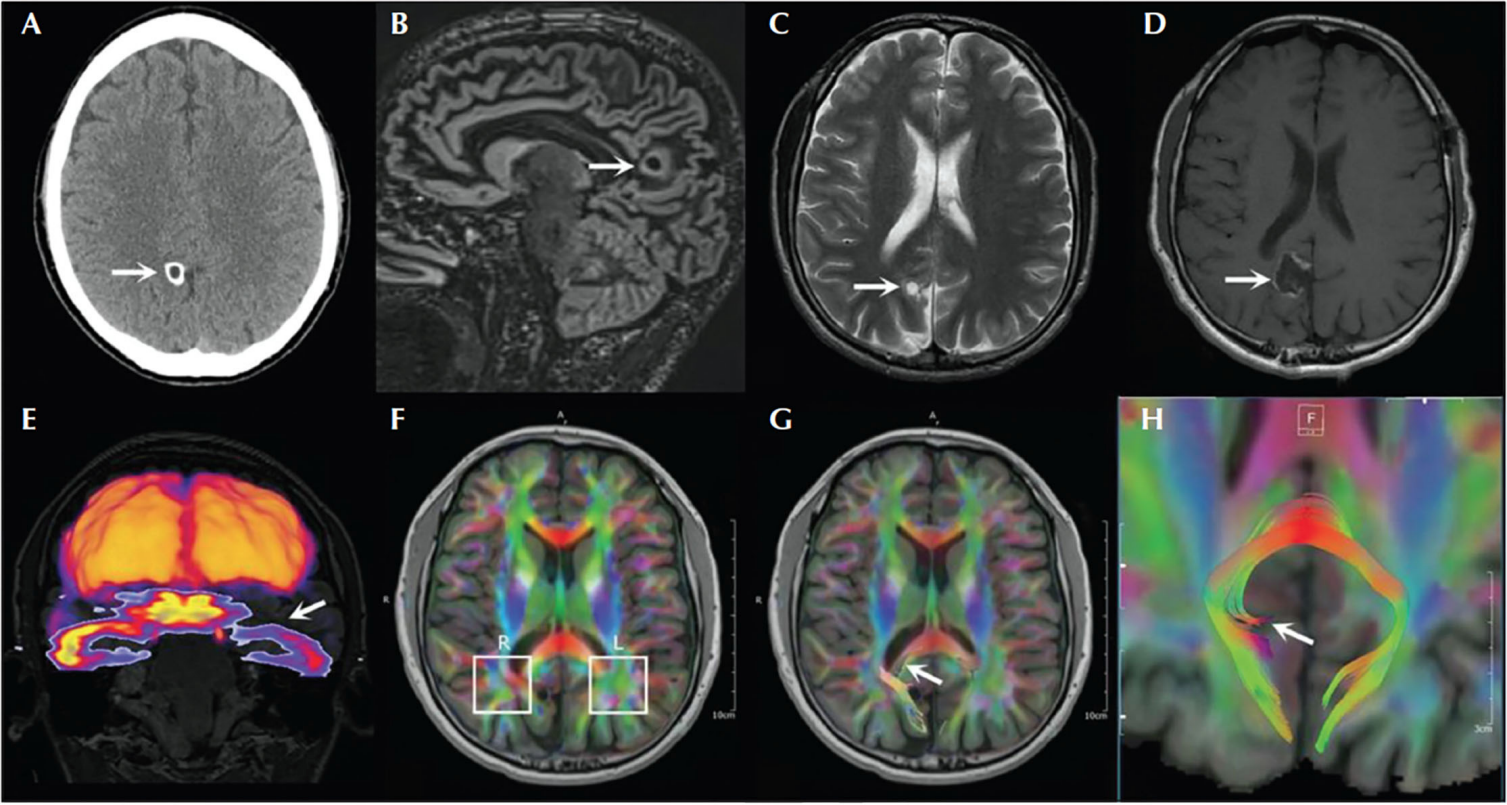
Brain imaging of the patient with right precuneus neurocysticercosis and left temporal lobe seizure involvement. **(A)** Head CT showing calcified lesion in the right precuneus. **(B)** Double inversion recovery MRI showing the lesion with surrounding gliosis. **(C)** T2-weighted image showing the lesion. **(D)** Postoperative MRI T1 sequence showing the cavity. **(E)** Coregistration of FDG-PET with MRI showing hypometabolism in the left temporal lobe. **(F–H)** Diffusion tensor imaging through the level of the lesion showing asymmetrical findings with respect to the parietal white matter [adapted from (8)].

Scalp video-EEG recordings captured four focal impaired awareness seizures (FIAS) with bilateral blinking at onset, left hand automatisms, sighing, right facial clonic twitching, and right arm tonic posturing for few seconds. The ictal EEG showed delta range discharges over the left temporal region which evolved into a sharply contoured rhythmic discharge in the theta range. Brain MRI showed a 0.7-cm cystic cortical lesion in the right precuneus with some calcification that was consistent with neurocysticercosis (Figure 1). FDG-PET showed left anterior temporal hypometabolism in addition to the expected hypometabolism in the right precuneal lesion. Furthermore, Abnormal peri-lesional tracts were evident on diffusion tensor imaging.

**Figure 2:**
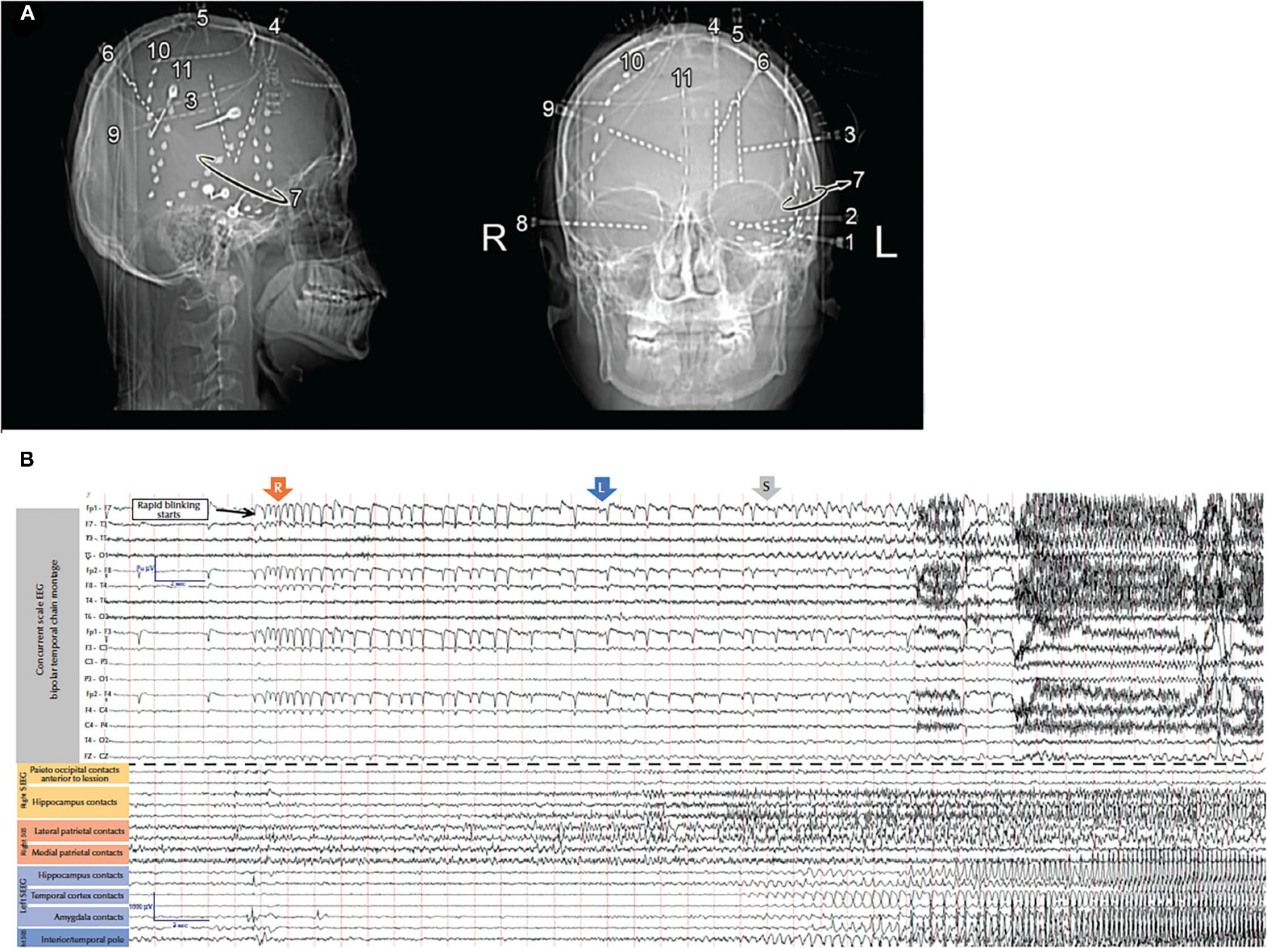
**(A)** X-rays showing the locations of the intracranial electrodes. **(B)** Scalp (top) and intracranial (bottom) electrodes in the amygdala, hippocampus, insula, and posterior cingulate gyrus in addition to the strips sampling right parietal lesion. The EEG shows onset around the right parietal lesion with secondary propagation to the contralateral temporal lobe [adapted from (8)].

Thus, while the seizure semiology, ictal EEG, and FDG-PET scan suggested left temporal lobe epilepsy, the MRI revealed a right precuneus lesion, which prompted invasive monitoring. SEEG and subdural strip electrodes targeting the left temporal lobe and the right parietal lesion were implanted. Seizures were captured with left hand automatisms, sighing, confusion, and postictal fatigue. Intracranial EEG showed spiking and low voltage fast activity in the medial and lateral parietal contacts before the ictal discharge propagated to the left mesial temporal structures and lateral temporal areas (Figure 2). Resection of the right precuneus lesion resulted in prolonged seizure freedom. Therefore, the seizure semiology, ictal EEG, and FDG-PET were all falsely lateralizing because they resulted from propagation to the contralateral temporal lobe. Indeed, the FDG-PET hypometabolism is known to occur not only in the seizure on zone but also in seizure propagation regions (21).

The PCC is another structure that is part of the default mode network and has close connectivity with the temporal lobe (17, 22). Located in the medial portion of the inferior parietal lobe, the PCC is inferior to the precuneus and superior to the corpus callosum (13). CCEP studies described robust connectivity of the PCC with various cortical and subcortical structures, especially the mesial temporal structures (14, 23). Tract tracing studies in non-human primates delineated bi-directional connectivity between the PCC and MTL structures (13). Furthermore, diffusion tensor imaging (DTI) enabled identification of direct connections between the PCC and MTL (24).

## Illustrative Case 2: (9)

This was a 22-year-old right-handed man with a 1-year history of FIAS and occasional focal to bilateral tonic-clonic seizures. The ictal manifestations were typical of TLE with staring, unresponsiveness, automatisms, and postictal confusion. His seizures also included left hand posturing. Despite being on five ASMs with therapeutic serum levels, he continued to have several seizures per month.

**Figure 3:**
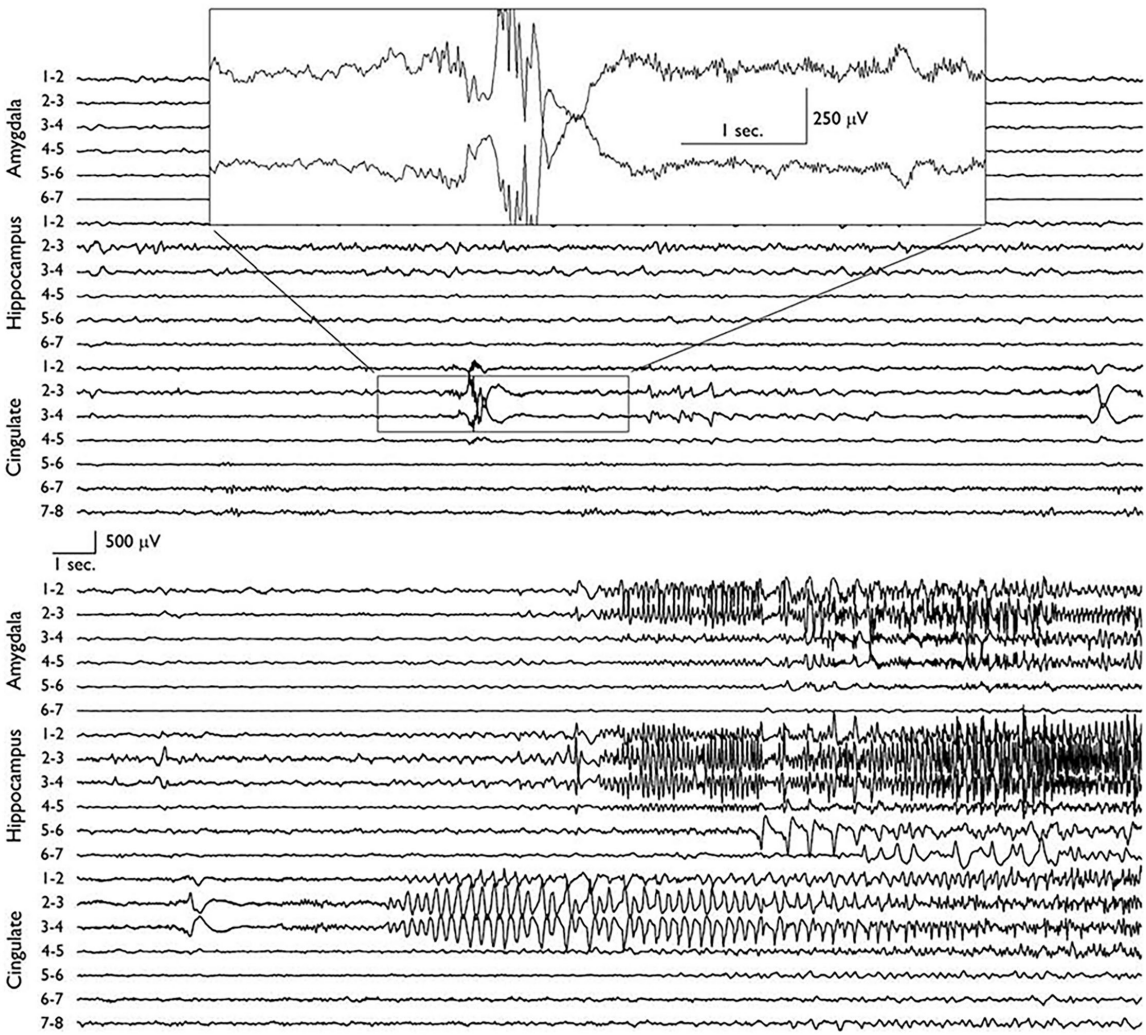
Depth electrode recordings of two consecutive EEG pages showing the ictal onset in the posterior cingulate area before later propagation to the ipsilateral mesial temporal structures. The seizure was semiologically silent before it involved the hippocampus and amygdala (9).

Scalp video-EEG revealed ictal discharges with a buildup of rhythmic theta-range activity over the right temporal derivations, typical of mesial temporal generators. This activity was in association with the typical aforementioned seizure semiology. Brain MRI revealed a round lesion of about one cm diameter in the right PCC, which was suggestive of dysembryoplastic neuroepithelial tumor (DNET). This prompted stereotactic electrode implantation targeting areas around the lesion as well as the mesial temporal structures. SEEG revealed that the seizures consistently started in the PCC as a low voltage, 40 Hz activity. After 20–30 s, the mesial temporal structures became involved with 6–8 Hz frequency ictal discharges seen over the hippocampus and amygdala (Figure 3). Only after propagation to the mesial temporal lobe did the seizure become clinically manifest. Resection of the DNET from the right PCC resulted in complete seizure freedom with a follow-up period of 2 years.

## Semiology

TLE, the most common form of focal epilepsy, may arise from mesial (hippocampal) or extrahippocampal neocortical sites, with the former accounting for about 80% of the presentations (25, 26). Mesial TLE predominantly presents as FIAS, and occasionally as focal aware seizures (FAS). Focal to bilateral tonic clonic seizures also occur in mesial TLE (7, 26). Auras have been reported to occur in more than 80% of patients with mesial TLE, and include viscerosensory (rising epigastric sensation), gustatory, olfactory, autonomic (piloerection, tachycardia, and flushing), experiential/psychiatric (déjà vu, jamais vu, auditory hallucinations, and depersonalization), mood changes (fear, agitation, and anger), and other phenomena (27, 28). Other common ictal manifestations include ipsilateral automatisms (lip-smacking/puckering), stereotyped gestures, contralateral head version, and contralateral tonic/clonic/atonic limb movements (29, 30). It is important to note that while a constellation of the aforementioned manifestations is highly suggestive of mesial TLE, each individual symptom is much less specific (31).

Parietal lobe epilepsy (PLE) is relatively uncommon, accounting for about 5–6% of surgically evaluated pharmacoresistant epilepsies (32, 33). It is typically underdiagnosed due to the intricate interconnections of the parietal lobe with other brain structures, allowing for a myriad of presenting semiologies and unreliable diagnostic yields (34). The ictal-onset zone in PLE can be clinically and electrographically silent. Hence, seizures may sometimes become manifest only after propagating to a another brain region, such as the temporal, occipital, or frontal lobes. Therefore, PLE can masquerade clinically as other epilepsies and falsely localize on non-invasive EEG (9). Occasionally, the parietal seizure-onset zone may be active, displaying a typical PLE semiology. This sheds light on the importance of being familiar with the semiologies that may hint toward a parietal lobe involvement.

With the parietal lobe as the symptomatogenic zone, PLE usually presents as contralateral, somatosensory auras (painful dysesthesias, numbness, tingling), vertigo, visual illusions, and disturbance of body image (e.g., perceiving that the contralateral limb is moving, turning, or becoming weak) (33). Pure sensory symptoms, such as numbness or dysesthesias, may suggest primary somatosensory (SI) onset. The presence of painful sensory phenomenon may suggest middle cingulate onset or parietal opercular (secondary somatosensory, or SII) onset (35).

The precuneus is a good example of heterogeneity in semiology based on functional connectivity: gaze deviation and head turn due to frontal eye field connections; visual-spatial distortion due to visual cortical connections; memory and attention impairment due to default mode network connections (which mimics TLE in particular); and cognitive dysfunction due to prefrontal connections (36).

The PCC can also present with symptoms that are not typically thought to represent parietal localization, including dissociative and dyscognitive symptoms (19, 23). In one series, the most common presentations of PCC seizures were limb posturing, vocalization, or hypermotor activity, and this is thought to be due to spread to frontal regions including pre motor and supplementary motor areas, as well as orbitofrontal cortex (37).

Dissociative and dyscognitive symptoms can be especially misleading, as it is commonly understood that TLE tends to alter alertness awareness or cognition. However, these symptoms can also arise from the precuneus and PCC (23, 36). Hyperkinetic or motor seizures may originate from posterior parietal regions or temporal-parietal opercular regions, similar to frontal lobe seizures (38, 39). A contralateral hyperkinetic movement accompanied by ipsilateral dystonic features is highly suggestive of a parietal origin of seizures (38). Additionally, insular-opercular regions can also mimic frontal localization (with nocturnal hyperkinetic seizures or contralateral focal motor seizures) (40).

In summation, while extratemporal lobe seizures, like PLE, might provide semiological hints toward the localization, it is common that the first symptom occurs due to the spread, rendering semiological analysis ineffective. Thus, it is important for the epileptologist to integrate the semiological findings with other diagnostic modalities to arrive at the appropriate epileptogenic zone and optimize the surgical outcome.

## Neuroimaging

Neuroimaging is a cornerstone in the presurgical evaluation of patients with pharmacoresistant epilepsy. It can provide the epileptologist with great diagnostic insight by confirming or disputing the putative source of the ictal electroclinical manifestations. Adequate postsurgical outcomes are highly dependent on the correct delineation and resection of the epileptogenic zone. While the majority of surgical candidates have a brain lesion on MRI, some are non-lesional. A meta-analysis has concluded that the odds of seizure freedom following epilepsy surgery (temporal, extratemporal, and combined epilepsies) in an MRI positive epilepsy were about 2.5–2.8 times higher than in patients with normal MRIs (41). That said, a study has shown promising results when comparing MRI-positive and MRI-negative epilepsies in terms of SEEG seizure localization and surgical outcomes for various focal epilepsies. The authors appropriately localized the epileptogenic zone in 96 and 95% of MRI-positive and MRI-negative cases, respectively. Furthermore, seizure freedom was achieved in 55% of MRI-positive and 53% of MRI-negative cases (42).

FDG-PET and ictal SPECT are additional diagnostic tools that can optimize presurgical assessments, especially in MRI-negative epilepsy. A study that compared MRI, PET, and SPECT among patients with neocortical epilepsies reported the sensitivities of 65.5, 77.2, and 73.8%, respectively, for identifying seizure-onset zones. Including only extratemporal neocortical epilepsy yielded the respective sensitivities of 56.7, 70.7, and 63.6% (43). Clearly, these latter figures apply better to medial parietal seizures. Further comparison showed an overall concordance rate among the aforementioned modalities to be 41.7%. Additionally, PET and SPECT correctly identified 19 of 32 (59.4%) and 17 of 31 (54.8%) lesional cases, respectively, among patients with normal MRI (43). This study stresses on the importance of utilizing and combining all available modalities during presurgical assessment, it also highlights on the usefulness of PET and SPECT in patients suffering from non-lesional epilepsy. Further, repeated SPECT studies can be helpful in confirming seizure-onset zones or identifying multifocality of epileptogenic zones (44). It is worth mentioning that while putative seizure-onset zones may be identified by ictal SPECT, the results can be misleading.

Recently, computational analyses of multimodal MRI data have been utilized to increase the chances of detecting seizure-onset zones in non-lesional focal epilepsy (45). The computational analysis is developed in samples of patients with lesional epilepsy before application to non-lesional cases. Such approaches may be further refined to reveal covert abnormalities in MRI-negative focal epilepsy patients. Indeed, co-registration of MRI with FDG-PET, which constitutes the standard of care at numerous epilepsy centers, has been shown to increase the detection of focal cortical dysplasia in some patients (46). Further, 3D multimodality imaging (with anatomical and functional MRI, vascular imaging, among others) has been shown to alter surgical decisions in patients with medically refractory epilepsy in need for surgical evaluation (47). An active area of research currently is using machine learning methods to predict laterality in patients with MRI negative focal epilepsy (48, 49). While none of these studies is specific to the medial parietal lobe, they may be applied to any focal epilepsy, whether mesial temporal or neocortical.

## EEG Monitoring

Scalp EEG recording is limited in localizing parietal seizures. Thus, invasive EEG should be considered when an extratemporal localization is suspected based on non-invasive presurgical assessment or when TLE surgery has failed (3). In a cohort of patients with pharmacoresistant parietal lobe epilepsy reviewed over 30 years, 93% of patients had a parietal lobe lesion on MRI, whereas only 20% had interictal epileptiform abnormalities on scalp EEG, and only 25% had parietal ictal onset on scalp EEG (32). This highlights the importance for invasive monitoring in these patients.

In a series of six patients with precuneal epilepsy, four were clearly lesional on MRI and three of whom had intracranial EEG. These three patients underwent surgical resections with an Engel Class IA outcome in two of the patients. However, Class IV outcomes where described in the two non-lesional patients who had intracranial monitoring in this series (15). In another study, SEEG confirmed precuneal seizure onset in 10 subjects where auras consisted of body image disturbance, vestibular symptoms, asymmetric tonic seizures, and hypermotor seizures (50).

Another invasive EEG study in a series of pharmacoresistant extratemporal lobe epilepsy patients found that nearly 90% of patients had non-localizing ictal scalp EEG patterns with widespread bilateral ictal activity or obscured onset by muscle artifacts (37). In 13 of the 18 subjects, nearly 100 seizures were analyzed by either SEEG or SDG, using a median of 137 contacts with 3D reconstruction and observation of high frequency oscillations (HFO). Interestingly, ictal onsets were seen simultaneously with ripples in the cingulate epileptogenic zone and the hippocampus. Surgical resection outcomes were favorable (12 of 18 patients with Engel IA outcome, three with IIB outcome). Importantly, if only the hippocampi had been monitored with traditional depth or strip electrodes, the simultaneous cingulate onset would have been missed. Thus, this limited series suggests the value of invasive monitoring in improving outcomes in cingulate onset seizures.

In assessing the utility of SEEG in identifying parietal opercular seizures, two patterns of epileptogenic network organization have been described (51). One is a mesial/cingulate pattern, which is associated with hypermotor semiology, and the other a perisylvian pattern, which is associated with contralateral focal motor seizures. These networks required SEEG for characterization. Although electrode arrangement was individualized, typical coverage included insular-opercular, mesial and lateral frontal, and mesial and lateral parietal. Therefore, when such semiology is present prior to a temporal lobe ictal discharge on scalp EEG, the parietal opercular region should be sampled in invasive monitoring.

A series describing the efficacy of SEEG monitoring for PCC epilepsy was reported in seven subjects and ictal onsets were identified by SEEG in six of these patients (23). Scalp ictal onset patterns were widespread and non-localizing, but SEEG secured confident localization and lateralization leading to resection.

Fast ripples (250–500 Hz) and other HFOs, have been suggested as a useful pattern in identifying the epileptogenic zone (33). Resection of these in the parietal regions should help improve outcomes, though formal studies in parietal onset seizures are not available, considering its rare nature. It is the authors' opinion that multimodal invasive EEG is recommended—extraoperative SEEG or SDG analysis of ictal and interictal findings, with evaluation for the presence of HFOs to identify resection margins. Use of interictal EEG findings (either intra- or extra-operatively) to guide resection should be done with extreme caution, as it can be misleading. SEEG has low morbidity in experienced centers, and allows 3D mapping of the epileptogenic zone, which is especially useful for deep structures like much of the parietal lobe.

## Outcomes

Surgical failures in TLE cast doubt on the initial diagnosis suggesting extratemporal epileptogenic zones. In cases where patients present with semiology and scalp EEG suggestive of TLE, but with a parietal lesion, the parietal lobe should be considered as a possible seizure onset zone. It is important to note, however, that a positive MRI may serve as a red herring since not all lesions are epileptogenic. Indeed, even in cases of non-lesional TLE epilepsy, the posteromedial parietal lobe should be considered for sampling by intracranial electrodes.

A thorough analysis of all the pre-surgical data is necessary for optimization of surgical outcomes in patients presenting with non-lesional TLE. In evaluating the seizure semiology, the auras can be a potential determinant of localization of the seizure onset zone (see above). Discordant findings on neuropsychological assessment should also be critically evaluated, especially with extratemporal localization of deficits, lack of clear lateralization of language or memory, as well as intact memory ipsilateral to the suspected TLE focus. Generally, neuropsychological assessments in TLE show impairment in language, memory, and visuospatial function. On the other hand, patients with PLE often exhibit normal memory but have impaired visuospatial function and sensory and language (dominant PLE) deficits. Nevertheless, neuropsychological results may mimic those of TLE in patients with extratemporal lobe epilepsy due to the temporal lobe being a symptomatogenic zone as discussed earlier. Deficits that are due to such symptomatogenic zone involvement tend to resolve following resection of the seizure onset zones (52). In addition, consideration of extratemporal localization should be made if there is no clear ictal onset pattern on scalp EEG.

When discussing the importance of identifying the underlying epileptogenic zone and planning surgical intervention for epilepsy, it is important to review the adverse events associated the invasive procedures and surgery. With low rates of morbidity, SEEG and SDE have the following complications listed in descending order; (1) hemorrhage (1%), with the most common underlying etiology being intracranial hemorrhage; (2) infection (0.8%), including cerebral abscess and meningitis; (3) neurological deficits (0.6%); (4) inappropriate positioning of electrodes (0.6%); and (5) electrode or recording malfunction. With regards to indirect adverse events, prolonged SEEG/SDE monitoring leads to longer hospital stays which may expose patients to infections like pneumonia and urinary tract infections. Furthermore, the resultant prolonged immobilization may lead to deep vein thrombosis and pulmonary embolisms (53, 54).

Finally, complications arise in about 7% in all types of epilepsy surgery, a trend that may be more evident in the elderly (55). Patients undergoing TLE surgery have been reported to experience diplopia (cranial nerve deficits mainly in trochlear nerve), visual field deficits (mainly superior quadranopsia), anomia, hemiparesis, psychosis and ischemia, among other surgical side effects, post-operatively (56, 57). PLE surgery, on the other hand, has been reported to cause partial sensory loss and Gerstmann syndrome (57, 58).

## Conclusions

In summary, applying knowledge of key semiological features and functional connectivity can identify further management options in presurgical TLE patients or ones who have already failed surgery for TLE. Semiology can be helpful but must be interpreted with caution, given widespread connectivity of the parietal lobe to other regions. In pharmacoresistant epilepsy, especially past surgical failures, it seems that imaging (especially MRI) has the most utility in localization over semiology and non-invasive EEG. Although data are limited, multimodal analysis can help epileptologists more confidently identify and localize parietal onset seizures, potentially leading to more successful patient outcomes. Newer medications, resection, and neurostimulation should remain viable options in such cases, even if surgery failed previously.

## Author Contributions

MZK conceived the idea of the manuscript. All authors contributed to the manuscript and NJ finalized the first draft and responded to the reviewers. All authors read and approved the manuscript.

## Conflict of Interest

The authors declare that the research was conducted in the absence of any commercial or financial relationships that could be construed as a potential conflict of interest.

## Publisher's Note

All claims expressed in this article are solely those of the authors and do not necessarily represent those of their affiliated organizations, or those of the publisher, the editors and the reviewers. Any product that may be evaluated in this article, or claim that may be made by its manufacturer, is not guaranteed or endorsed by the publisher.
